# Deciphering Tumour Heterogeneity: From Tissue to Liquid Biopsy

**DOI:** 10.3390/cancers14061384

**Published:** 2022-03-08

**Authors:** Pauline Gilson, Jean-Louis Merlin, Alexandre Harlé

**Affiliations:** Service de Biologie Moléculaire des Tumeurs, Institut de Cancérologie de Lorraine, CNRS UMR 7039 CRAN-Université de Lorraine, 6 Avenue de Bourgogne CS 30519, 54519 Vandœuvre-lès-Nancy, France; jl.merlin@nancy.unicancer.fr (J.-L.M.); a.harle@nancy.unicancer.fr (A.H.)

**Keywords:** tumour heterogeneity, circulating tumour DNA, liquid biopsy, treatment resistance, next-generation sequencing, multi-region sampling, single-cell approaches

## Abstract

**Simple Summary:**

Most malignant tumours are highly heterogeneous at molecular and phenotypic levels. Tumour variability poses challenges for the management of patients, as it arises between patients and even evolves in space and time within a single patient. Currently, treatment-decision making usually relies on the molecular characteristics of a limited tumour tissue sample at the time of diagnosis or disease progression but does not take into account the complexity of the bulk tumours and their constant evolution over time. In this review, we explore the extent of tumour heterogeneity and report the mechanisms that promote and sustain this diversity in cancers. We summarise the clinical strikes of tumour diversity in the management of patients with cancer. Finally, we discuss the current material and technological approaches that are relevant to adequately appreciate tumour heterogeneity.

**Abstract:**

Human solid malignancies harbour a heterogeneous set of cells with distinct genotypes and phenotypes. This heterogeneity is installed at multiple levels. A biological diversity is commonly observed between tumours from different patients (inter-tumour heterogeneity) and cannot be fully captured by the current consensus molecular classifications for specific cancers. To extend the complexity in cancer, there are substantial differences from cell to cell within an individual tumour (intra-tumour heterogeneity, ITH) and the features of cancer cells evolve in space and time. Currently, treatment-decision making usually relies on the molecular characteristics of a limited tumour tissue sample at the time of diagnosis or disease progression but does not take into account the complexity of the bulk tumours and their constant evolution over time. In this review, we explore the extent of tumour heterogeneity with an emphasis on ITH and report the mechanisms that promote and sustain this diversity in cancers. We summarise the clinical strikes of ITH in the management of patients with cancer. Finally, we discuss the current material and technological approaches that are relevant to adequately appreciate ITH.

## 1. Tumour Heterogeneity: From Historical Perspectives to Novel Insights

### 1.1. Varying Degrees of Tumour Heterogeneity

**Figure 1:**
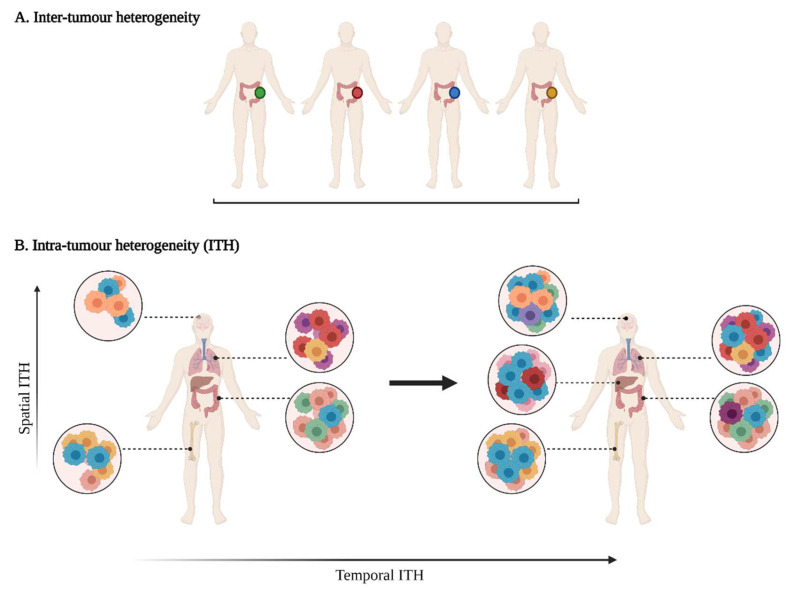
A multifaceted heterogeneity in cancers. (**A**) Inter-tumour heterogeneity refers to the variability observed in tumours of the same histological subtypes between different patients. (**B**) Intra-tumour heterogeneity (ITH) is observed across different regions of the primary tumour site and/or metastatic sites (spatial ITH) and can evolve over time (temporal ITH). Colours represent the different characteristics between tumours or tumour cells.

Tumour heterogeneity harbours multiple layers of complexity in human malignancies. It has long been known that tumours of the same histopathological subtype commonly differ from one patient to another (inter-tumour heterogeneity) (Figure 1). Exacerbating the complexity even further, sizable variations have been reported within a single tumour (intra-tumour heterogeneity, ITH). ITH can be detected between the different geographic regions of the same primary tumour or even between the primary tumour and the metastastic lesions (spatial intra-tumour heterogeneity). Moreover, the analysis of serial tumour samples demonstrated that the cell features may evolve during the course of the disease progression (temporal heterogeneity) under environmental or therapeutic stress [1,2]. ITH has been observed in most (nearly all) types of cancers, including both haematological malignancies (chronic lymphoblastic leukemia and acute lymphoblastic leukemia), and solid tumours (lung, breast, ovarian, pancreatic, kidney, colorectal, brain and prostate cancers) [3].

#### 1.1.1. Phenotypic Heterogeneity

**Figure 2:**
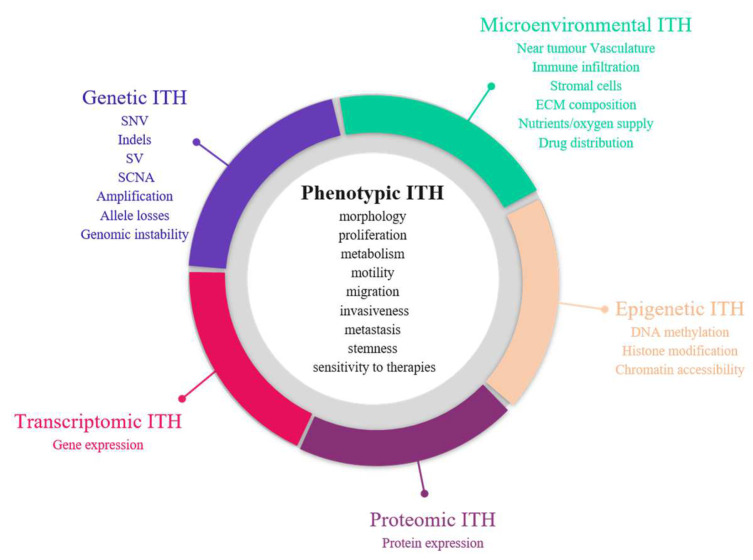
Sources of intra-tumour heterogeneity (ITH). Genetic, epigenetic, microenvironmental, transcriptomic and proteomic heterogeneities coexist in tumours and are linked with phenotypic diversity. Abbreviations: CAF: Cancer-associated fibroblasts; ECM: extracellular matrix; EMT: epithelial-to-mesenchymal transition; indels: small insertions and deletions; LOH: loss of heterozygoty; MET: mesenchymal-to-epithelial transition; SCNA: somatic copy number alterations; SNV: single nucleotide variants; SV: structural variants.

The first demonstration of tumour heterogeneity has been made by histopathologists who are familiar with morphological divergence (differentiation status, necrosis, fibrosis, etc.) across the tumours or between the different areas of the tumour (Figure 2) [1,4,5,6,7,8]. This notion has led to the very basis of tumour classification systems based on histopathological features [9]. Tumour grading systems notably include the pathological examination of multiple microscopy fields in order to avoid tumour misclassification due to ITH [10]. Increasing evidence indicates that tumour foci are heterogeneous at other phenotypic levels than merely morphologic, including differential capabilities in terms of proliferation, metabolism, motility, migration, invasiveness, metastasis and stemness, as well as varied sensitivity to therapies [11,12,13]. The morphological and other phenotypic cell features co-vary in the different tumour regions, notably between the core and the external borders of the tumour.

#### 1.1.2. Molecular Heterogeneity

Advances in next-generation sequencing (NGS) revealed the extent and prevalence of molecular diversity in tumours [14,15]. The sequencing of multiple regions in space and time demonstrated the various repertoires of genetic events that can occur in cancers, including single nucleotide variants (SNVs), small insertions and deletions (indels), structural variants and somatic copy number alterations (SCNA) [15]. Large-scale studies indicated that genetic ITH occurs in almost all cancer types, albeit at varying degrees [16,17]. Melanoma and lung adenocarcinomas notably account for cancers with high mutational tumour burden and the establishment of specific mutational signatures as a result of exposure to exogenous mutagens (UV radiation and tobacco smoke) [18]. Dietz et al. demonstrated that the frequencies of driver gene mutations in regionally distinct areas of lung adenocarcinomas were correlated with the spatial distribution of histological patterns, highlighting an interplay between histologic and genetic features in a tumour [19,20].

However, the genetic perspective is insufficient to fully explain the range of phenotypic diversity in solid malignancies, given the fact that cell populations with identical genetic background can lead to distinct morphological patterns and differential responses to treatment or environmental stimuli [10,21]. Emerging evidence demonstrated that ITH also take place at other levels, such as epigenetics, transcriptomics and proteomics [22].

The epigenome is defined as a connection between the genome and the environment. Alterations of the epigenetic machinery has been recognised as a hallmark of cancer [23] and may appear early during carcinogenesis [24]. Epigenetic marks induce heritable changes in gene expression without any modification in the underlying DNA sequence that allows cells to adapt to microenvironment stimuli (oxygen, nutrient deprivation, acidity, etc.) and develop resistance mechanisms against anticancer therapies [25]. Bidirectional communications between genetics and epigenetics have been reported in cancers, with the detection of somatic mutations in genes encoding epigenome regulators (such as *DNMT3A, IDH1, H3F3A*) and inversely the identification of DNA hypomethylation or epigenetic silencing of DNA repair genes (such as *MLH1* or *BRCA1*) that can cause genomic instability in cancer cells [24]. Studies assessing histone modifications, chromatin accessibility and DNA methylation profiles demonstrated a high epigenetic variability in cancers [21,26,27]. Considering the major implications of epigenetics in the development of cancers and their response to anticancer treatments, a better understanding of epigenetic heterogeneity could help to identify novel epigenetic therapies and consider them for a combination with other anticancer treatments (genotoxic/cytotoxic agents, hormone therapy, immunotherapy, targeted therapy) to improve their efficacy or reverse drug resistance [25].

Transcriptome refers to all RNA species that can be found in cells; however, mRNAs are frequently the most studied. Their composition varies between cell types and tumour types and continuously evolves depending on the local conditions that are applied to cells over time. They can be explored through targeted (RT-PCR) or high throughput approaches (gene expression arrays, RNA sequencing (RNASeq)). A plethora of gene expression signatures have been developed in oncology for tumour classification [28], prognosis establishment [29,30,31,32], therapeutic and surveillance decision making [33] but only a few are already implemented for routine practice [34].

Because proteins directly reveal the functional mechanisms that occur in cancers and account for most of the therapeutic targets, it appears important to assess tumour heterogeneity at the protein level, which has shown growing interest. Proteomic approaches have long lagged behind those for transcriptome and genome due to technical limitations, high amounts of proteins generated from a single gene (with different isoforms and modification states) and a complex regulation of protein expression at both translational and post-translational levels [35]. Immunohistochemistry appears as one of the most standard approaches to assess protein abundance changes; however, it provides only semi-quantitative information, interrogates a limited number of proteins and is limited by the availability of appropriate antibodies. The development of reverse-phase protein array (RPPA) and mass spectrometry (MS)-based methods enabled the assessment of the proteomic landscape on a larger scale [35]. Transcriptomic approaches cannot substitute proteomic investigations, as the analysis of datasets from The Cancer Proteome Atlas (TCPA) found a poor correlation between protein and gene expression in cancer tissues, with Spearman correlation oscillating from 0.1 to 0.3, depending on the cancer type [36].

Recognition of all these cancer-specific molecular processes as major elements in the evolution of cancers and their considerable diversity has led to the launch of cancer genomics programs by international consortia, such as The Cancer Genome Atlas consortium (TCGA) and the International Cancer Genome Consortium (ICGC). All aim to integrate the analysis of multi-omics datasets (genetic, epigenetic, transcriptomic and proteomic data) to provide a comprehensive overview of the tumour landscape [16,37,38,39,40,41,42,43,44,45].

#### 1.1.3. Tumour Micro-Environment (TME) Heterogeneity

The influence of the complex ecosystem in which cancer cells evolve has long been overlooked. In the last decade, cancer biology progressively shifted from a cancer cell-centric model to a more ample view, where cancer cells and their near environment are highly interrelated. The tumour microenvironment (TME) is made up of non-transformed cells (endothelial cells, fibroblasts, pericytes, adipocytes, immune cells, etc.) and non-cellular constituents (such as the extracellular matrix) which are shaped by cancer cells through the modification of local environmental conditions and the secretion of oncogenic signals [46,47]. As a consequence, the phenotypic traits and behaviours of TME components are highly heterogeneous, depending on the tumour context (Table 1) [46,48]. In return, TME can assist in the development of the tumour niche by contributing to cancer progression, metastasis and drug resistance [48,49,50]. In this way, TME represents an emerging target for treatments (such as immune checkpoint inhibitors or antiangiogenic therapies) and should be taken into consideration for clinical decisions. Recently, Garattini et al. demonstrated that heterogeneity also extends to the drug distribution in tumours, which depends on many aspects of the patient, the tumour and its microenvironment and influences tumour response [51].

### 1.2. Unravelling Evolutionary Processes behind Tumour Heterogeneity

Two major and paradoxical theories have been developed to explain the installation of high degree of diversity in tumours. In 1976, Peter Nowell first described the cancer development as a continuous evolutionary process originating from a single renagade mutant cell and driven by the accumulation of stepwise somatic mutations during proliferation processes that give rise to various clones and subclones [61]. The development of multiple cell groups with distinct genomic profiles is amplified by genomic instability that arises in most solid tumours and haematopoietic malignancies as a result from both exposure to exogenous mutagens and defects in DNA repair pathways [62]. A subclone is characterised as a set of cells that diverge from the cell ancestor lineage (clone) by the presence of additional genetic alterations. Equivalent to Darwinian natural selection, most stochastic events that appears during the evolution process probably do not confer any selective benefit to the cancer cells (passenger mutations) [63]. In contrast, certain mutations can provide a fitness advantage over adjacent cells (driver mutations) and enables them to become predominant and outcompete other ones [64]. Most driver mutations are clonal. They appear early during cancer progression under a given microenvironmental context and foster cancer progression but they seem not essential for cancer maintenance once installed [63]. The clonal genomic architecture is distinct from a tumour to another considering that the emergence of subclones strongly depends on specific environmental stresses (local hypoxia or inflammation, treatment exposure, etc.) applied in each tumour over time. More recently, epigenetics and genetics were shown to follow convergent evolutionary trajectories in the development of cancers, highlighting the potential interest of combining epigenetic agents with other anticancer therapies [65,66,67].

In contrast to the Darwinian clonal evolution theory, where all subclones possess tumorigenic potential, a second model proposed that only a small subgroup of cancer cells (named cancer stem cells (CSC) or tumour-initiating cells) has the capacity to generate new tumours [68,69]. In this model, tumours are structured in a unidirectional hierarchy fashion, whereby CSC can either indefinitely self-renew (symmetric division) or differentiate into multiple cancer cell types (asymmetric division). CSC with stem cell-like characteristics have been observed in several cancers, including leukemia, breast, colon, head and neck and oesophageal cancers [64]. CSC are thought to be more drug-resistant than non-CSC and in such ways, they may be responsible for recurrence and therapeutic evasion [68]. Increasing evidence, however, indicated that non-CSC can readily convert to a CSC state through cell plasticity programmes, such as epithelial-to-mesenchymal transition (EMT), indicating that the hierarchy seems less rigid than previously thought [70]. In the same manner, different subsets of CSC with variable EMT phenotypes can coexist in tumours and can switch from one to another [70]. Stemness and CSC plasticity may be modulated by internal (genetic and epigenetic) and external (TME) factors that can work apart or simultaneously [71]. Van Niekerk and colleagues show that certain stem cell features can be acquired by cancer cells through clonal selection, highlighting the fact that clonal evolution and the CSC theories are not necessarily mutually exclusive and can intertwine [72].

**Figure 3:**
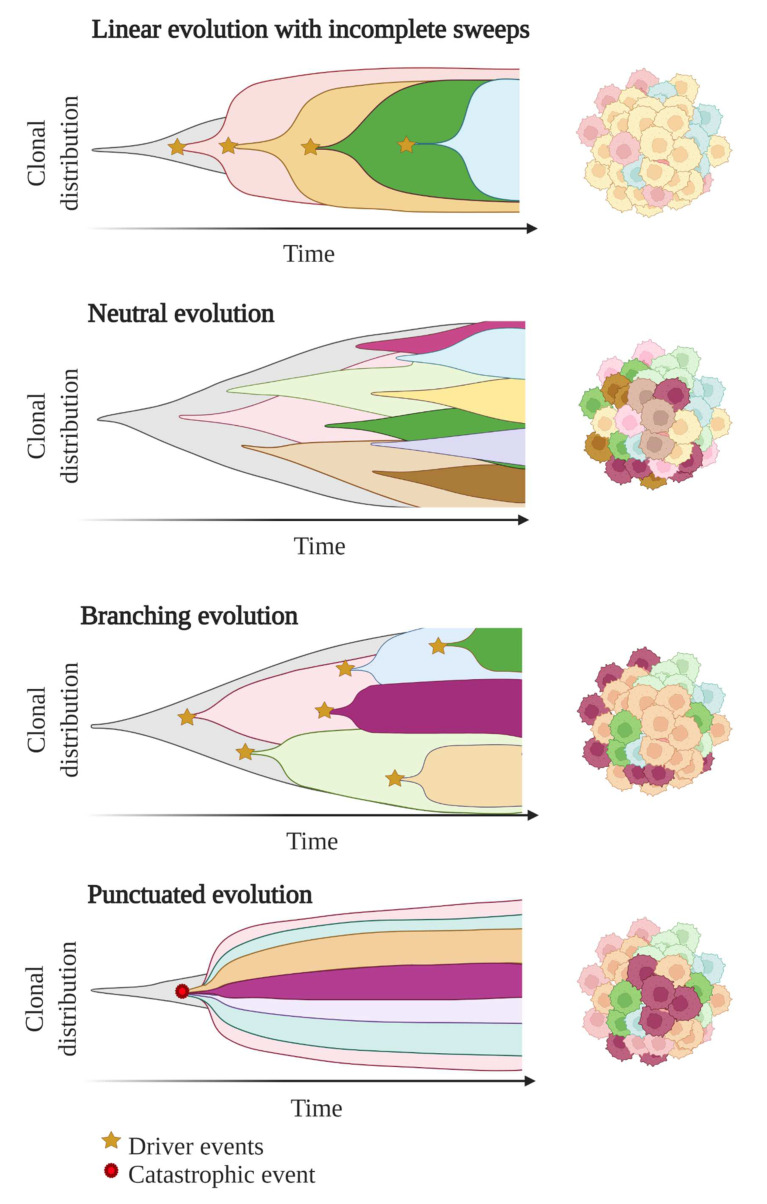
Models of tumour evolution described by Muller plots, which represent the tumour clonal dynamics over time. Colours indicate the different genotypes of the tumour cell clones.

In these first models of tumour evolution, ITH was thought to gain gradually over time as the tumour grew (Figure 3). Although this concept of continuous clonal evolution is still applicable to describe most cancer evolutionary processes, increasing evidence supported the idea that this model cannot explain the full spectrum of observed evolutionary behaviours [73]. Notably, single catastrophic events, such as whole-genome doubling, chromosomal chromoplexy and chromothripsis, can arise suddenly as single macroevolutionary jumps over long periods of relative stasis. In some extreme cases of punctuated tumour evolution, the development of colorectal cancers and other tumour types has been modelled as “Big-bang” dynamics, whereby a single or few mutational bursts occur early during carcinogenesis and result in a large number of intermixed subclones that are not subjected to selective pressure and coexist during growth (neutral evolution) [74,75,76].

Branched evolutionary trajectories have been extensively described in a wide range of tumour types, such as childhood acute lymphoblastic leukaemia, clear cell renal carcinomas, pancreatic, colorectal, breast and prostate cancers [77]. Evolutionary pathways can then be represented as a phylogenetic tree, where truncal mutations (clonal) represent the alterations occurring early in cancer development progenitors, while nontruncal mutations (subclonal) emerge during cancer progression and are shared by only a small group but not all cancer cells. In a branched evolution pattern, several distinct subclones co-exist and can be either intermingled in the same area or regionally separated, depending on the presence of physical barriers, such as blood vessels or microenvironment specificities [62].

ITH has also been described in cancer cases with linear evolutionary trajectories whereby a predominant subclone outgrows at the expense of its predecessor(s) followed by incomplete selective sweeps [62,77]. Although most studies described a single model of evolution in cancers, emerging data suggest that tumours may follow different models of evolution (linear, branched, punctuated or neutral) sequentially or simultaneously during the course of the disease [78]. The full context of tumour evolution is still to be explored in detail in order to better define effective therapeutic strategies.

## 2. Clinical Consequences of Tumour Heterogeneity

### 2.1. Impact on Diagnosis, Prognosis and Therapeutic Predictions

In the last decades, the management of patients with cancer has been revolutionised by better knowledge on the molecular background in cancer development. The understanding of molecular inter-tumour heterogeneity has formed the basis of personalised medicine in diagnosis, prognosis and treatment of cancers. Notably, it has set the limits of using universal anticancer drugs and has been a major driver for the emergence of novel therapies targeting specific molecular characteristics [79]. For most cancers, molecular diagnosis has entered in clinical practice as a prerequisite for tumour subtyping, prognosis refinement and treatment-decision making. Molecular testing is routinely performed on a limited tumour tissue area selected by the pathologist to be the most representative of the tumour. However, such methodology induces inherent under-sampling bias due to spatial and temporal ITH.

#### 2.1.1. Tumour Sampling Bias Due to Spatial ITH

Most of the histopathological and molecular features are not expressed homogeneously in tumour subpopulations, highlighting the fact that the analysis of a single sample may lead to diagnostic and prognostic errors and provide an incomplete view of potential vulnerabilities to treatment [80]. The identification of an actionable mutation in a predominant subline might not necessarily predict the response of the bulk tumour [79]. If the targeted mutation is shared by only a subset of cancer cells in the tumour (nontruncal mutation), the response to the targeted therapy is often of limited duration due to the outgrowth of resistant pre-existing subclones and/or the development of new drug-tolerant clones under therapeutic selection pressure [81]. In patients with metastatic disease, it is of clinical importance to portray ITH, given the fact that a genetic shift is infrequently observed between a metastase and the primary tumour site or even between two spatially distinct metastases [2].

#### 2.1.2. Tumour Sampling Bias Due to Temporal ITH

Archival tissue specimens commonly serve as starting material for testing if any recent sample is available. These samples can be collected many months or years previously, at the time of diagnosis or when a new lesion appears at distance. However, they cannot reliably reflect the tumour landscape over the time, considering that the tumour constantly evolves under specific microenvironmental conditions (such as acidosis, hypoxia or reactive oxygen species) [82] or exposure to therapeutic lines (DNA damaging agents or radiotherapy, targeted therapy and immunotherapy) [83]. In this context, treatment failure may happen when therapy is directed against a specific molecular characteristic [10].

#### 2.1.3. Determining ITH to Decipher the Identity of the Tumour or of Specific Regions of the Tumour

Analysing the characteristics of a tumour or of different tumour regions allows us to define the tissue of origin but also provide insights into the molecular events that occurred sequentially or in parallel throughout the development of the tumour. In this context, evaluating ITH gives a remarkable view of the whole history of the tissue and could help to better understand cancerogenesis and develop new therapeutic strategies.

#### 2.1.4. ITH Is Associated with Poorer Clinical Outcomes

The analysis of data collected by the TCGA from more than 3300 tumours across nine tumour types revealed that ITH has prognostic utility [84]. High degree of ITH are closely related to poorer immune infiltration and worse prognosis for patients with solid malignancies, including head and neck carcinomas, glioma, melanomas urothelial, breast, renal, lung and prostate cancers [84]. The relationship between ITH and patient outcomes is, however, complex to interpret and can be influenced by many aspects of the tumour, including the tumour cell of origin, the number of clones, the level of chromosomal instability, the type of somatic events and their order of appearance [85]. The prognostic value of ITH concerns other aspects than just genetic diversity [86,87,88,89], suggesting the importance of capturing the full extent of ITH.

#### 2.1.5. High Amounts of Biomarkers to Analyse in Order to Fully Decipher ITH

Emerging omics technologies have shown their interest in recent studies to analyse all types of ITH. However, multi-omics analyses are still far from standard-of-care, considering their cost, their low spread in clinical labs and the need for powerful data storage options for these big data. The analysis of only few histomolecular biomarkers in tumours is still the reference and may lead to misinterpretation.

Considering the huge amounts of data generated from multi-omics approaches and their high complexity, there is a considerable need to develop automation tools able to provide an integrative analysis of the multiple layers of heterogeneity without any expert intervention. Last advances in artificial intelligence and machine learning models allowed us to better predict cell subtypes and infer their proportions in tumours and TME based on their inherent multi-omics characteristics. These approaches have the potential to integrate both molecular and histopathological imaging data to refine tumour heterogeneity in the spatial context and go beyond what can be distinguished by routine microscopy observations [90,91]. However, due to their recent development, they still lack standardisation and need further evaluation prior to their implementation in a clinical setting [91].

### 2.2. Impact on Therapeutic Strategies

Although the notion of ITH and its impact on therapeutic response is now well documented in research studies, ITH determination is rarely taken into account in current clinical decision making that mostly relies on short-term treatment efficacy and the detection of resistance mechanisms to adapt the treatment and forestall disease relapse. However, capturing ITH could aid in developing novel strategies to provide long-term drug response and minimise the emergence of resistance mechanisms [83]. For example, in cases of heterogeneous tumours, upfront combination of therapies targeting different cancer cell subpopulations or dependencies could help to obtain a more durable response by minimising ITH and hindering minor subclones to expand under monotherapy pressure. Given the high molecular diversity that can be observed between the different regions of tumours, targeting all alterations is clearly unrealistic in clinical practice. The development of such strategies would require the determination of the aberrations the most critical for cellular functions and survival beforehand. The use of targeted therapy associated with non-targeted agents or ITH-reducing agents (such as histone deacetylase (HDACi), bromodomain and extra-terminal protein (BETi) or histone demethylase (HDMi) inhibitors) could also be considered to prevent the emergence of resistance in the case of highly heterogeneous distribution of a molecular target in tumour tissues [83]. Some groups also proposed adaptive therapies as a way to stabilise the balance between drug-sensitive and drug-resistant subclones and maintain tumour burden [92,93,94].

## 3. Emerging Approaches to Evaluate ITH

**Figure 4:**
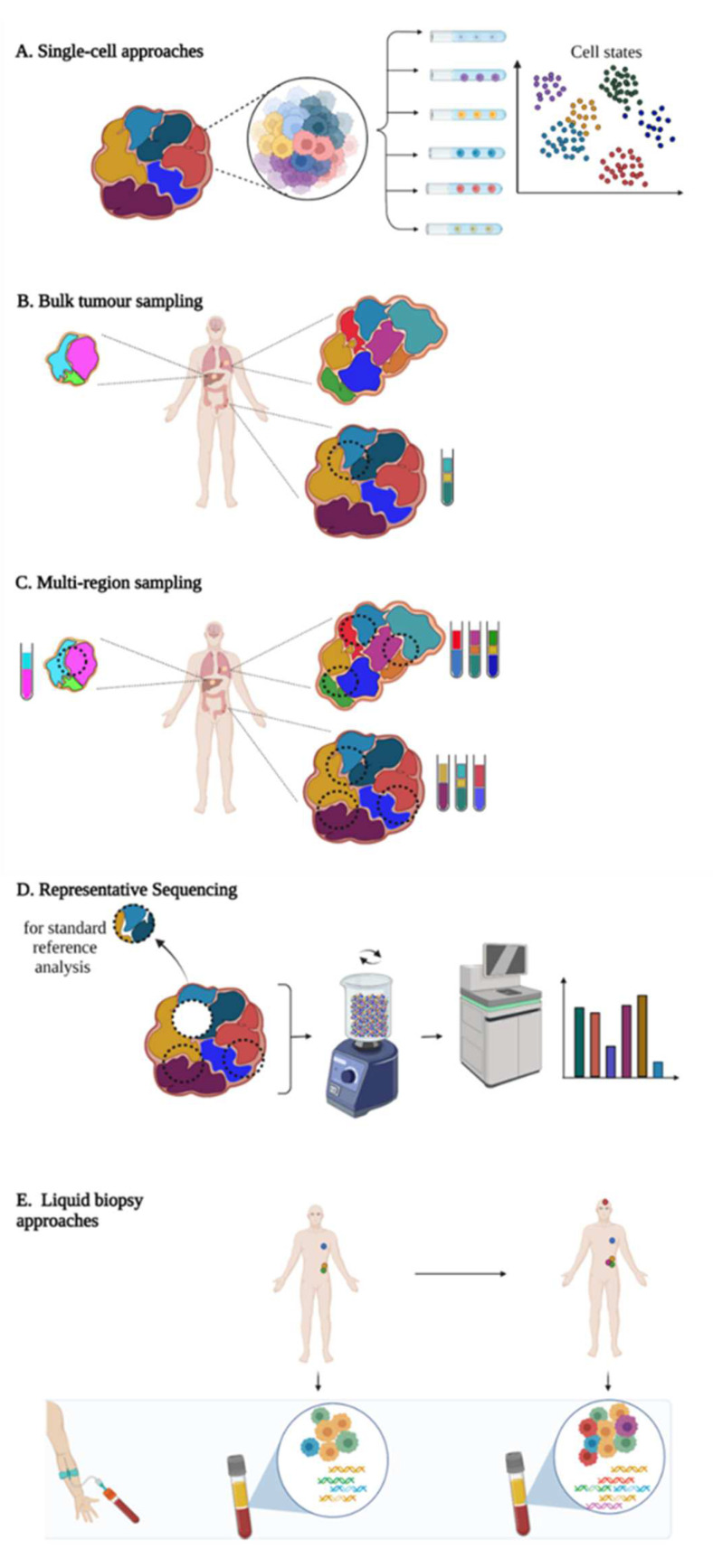
Emerging approaches to capture intra-tumour heterogeneity (ITH). (**A**) Single-cell approaches profile the characteristics of each cell individually. Compared to bulk-tumour sampling (**B**), multi-region sequencing (**C**) measures cellular heterogeneity in distinct regions of the primary site and of the metastases and/or tissue biopsy samples collected at different time points of the disease. (**D**) Representative sampling (Rep-Seq) implies the analysis of a mixed solution composed of tumour residual material that has not been used for standard pathologic procedures. (**E**) Liquid biopsy consists in the analysis of tumour-derived components shed in body fluids.

Decoding tumour heterogeneity is a major clinical challenge, considering that it immensely contributes to cancer progression, treatment failure and emergence of drug resistance. Emerging technical and sampling strategies have been developed in order to deeply characterise tumour complexity and clonal architecture, including single-cell profiling, multi-region sampling, representative sampling and longitudinal analysis of liquid biopsy samples (Figure 4).

### 3.1. Bulk-Cell Versus Single-Cell Approaches

Most studies that aim at profiling ITH and clonal evolution were based upon standard bulk-cell methods [73], that refers to the analysis at once of all cell populations from a sample, whatever their characteristics. Such approaches are able to infer tumour phylogenies through computational methods. Briefly, the variant allele frequencies (VAF) of each somatic mutation are converted into cancer cell fraction (i.e., the proportion of cancer cells that carry the mutation), estimates that indirectly permit the determination of the tumour subclonal architecture by assigning the mutations into the different clones. However, the estimation of the cancer cell fraction can be biased by tumour purity and copy number state of the locus. Moreover, bulk-cell approaches, by nature, average the signals emanating from the different tumour cell subgroups and can miss some variants only present in minor subclones, which can be confounded with noise [73,95]. Finally, a high number of computational tools has been developed but their use is sorely lacking in standardisation of their methodologies [96].

Single-cell approaches (SCA), based on the analysis of a high number of cells individually, gained popularity in the last few years, as they can unambiguously reconstruct tumor subclonality. SCA provided a valuable opportunity to detect slight cell-to-cell variations and perform cell-type clustering, determine ITH and TME composition at high resolution level, and evaluate the cellular evolutionary relationships [97,98,99,100,101]. Single-cell RNA-sequencing (scRNAseq) accounts for one of the most applied technologies [102,103,104,105]. Through the assessment of gene expression patterns at the single-cell level, scRNAseq allowed the detection of rare residual cells after treatment or the identification of minor drug-resistant cell populations implied in disease relapse that would be missed by conventional bulk-cell methods [106]. Some scRNAseq approaches may also help to predict cellular evolutionary dynamics and future cell states [106]. scRNAseq protocols include a single-cell isolation step (by flow-activated cell sorting or microfluidic device, for example), followed by RNA isolation, reverse transcription and cDNA amplification before library preparatin, sequencing and bioinformatics analysis. cDNA amplification represents a critical step, as sequencing methodology requires more input than we could expect to isolate from a single cell; however, the use of unique molecular identifiers (UMIs) allows us to reduce PCR error rates. The elevated risk of false negatives with scRNAseq due to allelic drop-out or technical noise is challenging and requires the use of adapted pipelines to analyse the data [73,107]. Recently, multi-omics SCA have emerged as promising tools to capture different aspects of ITH and cancer evolution at once [106,108,109]. Contrary to separate single omics analyses performed on distinct cells, multi-omics SCA can undoubtedly establish the correlation between the different macromolecules and their dynamics without any confounding influence of ITH [108]. In this way, they reveal a more comprehensive view of the state of an individual cell. Although the cost of the single-cell NGS-based approaches has progressively reduced in the last years, it remains much more expensive than the bulk-cell options, as they need sequencing at higher coverage depth [110].

### 3.2. Tissue Biopsy Sampling Approaches

For decades, the tissue biopsy sample remains the reference practice for tumour testing. However, tissue biopsy sampling implies an invasive and putatively morbid procedure. Further, it may be unfeasible in certain cases, as the tumour site is not accessible. Moreover, the analysis of a unique limited tumour tissue sample provides by nature only a spatiotemporal snapshot of the tumour. The input material for routine molecular characterisation generally represents as little as 0.0005% of the tumour bulk, making it inappropriate to fully capture the complexity of the whole disease [111].

Procuring multiple samples of the tumour for molecular analysis represents one way to circumvent this limitation [112,113,114]. Multi-region sampling refers to the use of multiple spatially distinct regions of the same tumour and of its metastases and/or tissue biopsy collected from the same patient at different time points [73]. Some studies already reported the interest of multi-site sampling approaches to better portray ITH and reconstruct the cancer evolutionary history in diverse malignancies, including lung cancers [115], clear-cell renal cell carcinomas [80,116,117], ovarian cancers [118], melanomas [119], osteosarcomas [120], urothelial carcinomas [121] and esophageal squamous cell carcinomas [122]. Notably, the TRACERx (TRAcking Cancer Evolution through therapy/Rx) project is a UK-based large-scale longitudinal observational study based on multi-region sampling that aims at charting evolutionary dynamics of some malignancies, such as melanomas, lung, prostate and renal cancers. As an example, the TRACERx renal cohort has served to interrogate the molecular landscape of clear-cell renal cell carcinomas (ccRCC) starting from the analysis by whole-exome sequencing of 1206 tumour regions from 101 patients. It demonstrated mutational (0–15 per tumour with a median of three per tumour) and copy-number (1–14 per tumour with a median of seven per tumour) ITH, to a higher degree than previously observed in single-biopsy studies [117]. The clone number per tumour was highly variable (ranging from 1 to 23) and strongly correlated with the stage and the grade of the disease. By analysing the timing of the somatic events in cancer evolution, the co-occurrence or contrariwise the mutual exclusivity of driver events in tumour subclones, Turajlic et al. identified seven distinct evolutionary trajectories associated with different phenotypic properties (Ki67 staining, levels of ITH and genome instability and clinical outcomes). To better understand the genetic evolution of metastases in ccRCC, subclonal heterogeneity was compared between paired primary and metastases regions from 38 patients of the TRACERx Renal cohort [116]. Metastases significantly display less driver events (nine mutations and/or SCNA in average) and were found more homogenous (13% of subclonal events) than the primary tumours (12 mutations and/or SCNA and 68% of subclonal variants, respectively). A total of 456 driver events were common in matched primary tumours and metastases, while 230 were found exclusively in primary sites and 39 exclusively in metastases. Based on the phylogenetic reconstructions of metastatic progression, Turajlic’s group distinguished three types of tumour clones: (1) clones present only in primary tumours that were “not selected” for metastatic dissemination, (2) clones “maintained” during metastatic seeding and shared between primary site and metastases and (3) clones “selected” for metastasis formation, that were found subclonal in primary tumour and clonal in metastases or appear de novo in metastases. Selected clones were characterised by elevated genomic instability, ploidy and proliferation index and HLA allelic imablance, compared to non-selected clones. Based on a meta-analysis performed on three distinct cohorts, loss of chromosome 9p or 14q is more frequently observed in selected clones compared to non-selected ones, suggesting their potential role in promoting metastasis. The timing of dissemination and the extent of metastatic seeding seem conditioned by the levels of ITH and genomic instability in primary tumours as well as the evolutionary subtype. Notably, progressive diseases were characterised by the presence of multiple clonal driver alterations, *BAP1* mutation or *VHL* wild-type status, lower ITH and high genomic instability. Contrariwise, attenuated progressive diseases (longer time of metastasis, single metastatic dissemination) preferentially harboured “*PBRM1* → *SETD2*” or “*PBRM1* → *PI3K*” evolutionary trajectories, high ITH and low genomic instability in primary tumours. This example illustrates well the interest of multi-region sampling to better decipher the impact of ITH and genetic evolution on cancer progression. However, such strategy seems impractical in routine care given the invasive nature of the process and the marginality of tissue rebiopsy during the disease. Moreover, the analysis of multiple samples in parallel implies tedious and costly procedures that cannot be easily performed on standard tumour biology platforms.

Pooling DNA/RNA extracts from multiple anatomically distinct regions of the tumour before sequencing could represent an option to reduce the cost, while optimising the variant detection rates as well as the determination of mutation prevalence and clonal clustering in the tumour mass compared to single-biopsy sampling [111]. Nevertheless, such strategy implies the identification and selection of the more representative tumour areas to biopsy with the non-negligible risk of missing a region of interest.

Representative sampling has been proposed by Litchfield et al. as a more realistic approach for clinical routine [111]. Representative sequencing (Rep-Seq) protocol consists in the analysis by next-generation sequencing (NGS) of a homogenised solution prepared from all residual tumour material that was not used for standard pathology practice. Rep-Seq methodology proved its higher reproducibility in detecting variants (95% of similarity between Rep-Seq replicates) in ccRCC compared to single-biopsy sequencing (78% of similarity). Further, it succeeds in detecting variants with frequency as low as 0.15%. VAF obtained by Rep-Seq correlated well with those found by pooling sequencing data generated from 64 spatially distinct primary biopsies (simulating the whole primary tumour). Rep-Seq accurately determined the clonal architecture of the tested ccRCC specimen compared to multi-region sequencing and avoided pitfalls of “clonal illusion” (detection of a mutation that seems clonal in the tested sample but is actually subclonal in the bulk tumour) that can be frequently observed with single-biopsy and multi-region biopsy sampling. In the context of metastatic melanoma, Rep-Seq performed on lymph node residual tissue was able to successfully determine the polyclonal tumour structure that was missed by single-biopsy analysis (results are, however, nuanced, in that Rep-Seq failed to detect some tumour subclones identified by multi-region sequencing). A proof-of-concept study confirmed the capacity of Rep-Seq to detect driver events in eight non-ccRCC cancer samples (breast, colorectal and lung).

### 3.3. Post-Mortem Samples

Emerging studies resorted to the analysis of tumour samples collected from patients during autopsy to tackle the encountered limitations of sampling in living patients [73,123]. Such an approach has the advantage to give access to high quantities of tumour material emanating from different organs, tissues or body fluids and can represent a useful strategy to study tumour evolution under therapeutic pressure and the emergence of resistant cancer cell clones [73]. Rapid research autopsies already demonstrated their interest in delineating ITH and tumour evolution (particularly in the context of treatment resistance) in several malignancies, such as lymphomas, cholangiocarcinomas, breast, prostate and lung cancers [124,125,126,127,128]. Growing efforts are made to launch large-scale post-mortem studies, such as the UK national PEACE (Posthumous Evaluation of Advanced Cancer environment) program (PEACE ClinicalTrials.gov number, NCT03004755). The setting up of rapid autopsy programs require the development of close collaboration between clinicians, researchers and the forensic team, the adaptation of autopsy logistics and procedures and the gathering of previous consent of the cancer patients and their family [73].

### 3.4. Liquid Biopsy-Based Approaches: A Better Reflection of ITH?

Liquid biopsy (LB), based on the analysis of tumour markers circulating in body fluids (including circulating tumour cells (CTCs), circulating tumour nucleic acids (ctDNA, ctRNA), circulating nucleosomes and tumour-derived extracellular vesicles), is emerging as a promising alternative to tumour tissue biopsy. Numerous studies already demonstrated its interest as a minimally invasive and easily repeatable tool for cancer screening, diagnosis, prognosis, treatment stratification, minimal residual disease (MRD) evaluation, disease monitoring and early detection of resistance mechanisms [129,130,131,132,133]. Plasma samples represent the most studied source of tumour material; however, recent studies highlighted the interest of other body fluids (including urine, saliva, stool, pleural effusions or cerebrospinal fluids) for clinical investigations in cancers [134]. LB is argued to better reveal tumour heterogeneity by randomly capturing the molecular alterations carried by the clones from all spatially distinct tumour regions. Moreover, the analysis of serial plasma samples seems easy to perform and could provide a non-invasive way to track the dynamic nature of cancers. Different tumour-derived components, especially ctDNA and CTCs, were assessed for their utility to decipher ITH.

#### 3.4.1. CTCs

CTCs have been less extensively studied than ctDNA but research studies on CTCs have been on the rise for a few years. CTCs were found to be heterogeneous at genetic and phenotypic levels and evolve over the time as the tumour progresses [135]. As CTCs can be shed by both primary sites and metastases, heterogeneity observed in CTCs could give a global overview of the molecular landscape of the whole disease at a fixed time [136,137]. Recent evidence reported a correlation between CTC count and cancer progression as well as the patient’s survival [135]. Beside its prognostic value, the analysis of CTC heterogeneity seems also of clinical significance to monitor tumour response, guide treatment decisions, predict drug resistance and unravel the mechanisms behind them [138,139,140,141,142,143,144]. Moreover, some groups reported the remarkable capacity of CTCs to form clusters (in association or not with non-malignant cells) that enrich ITH and favour CTC homing to distant organs (with a metastatic potential 23- to 50-fold higher compared to single CTCs) [135,145]. In this context, CTCs identification and characterisation could help to understand the biological processes implied in metastatic seeding.

CTC-based approaches imply a critical step of CTC isolation from blood samples prior to single- or multi-omics analysis (genetics, epigenetics, transcriptomics and proteomics). CTC analysis based on single-cell approaches has the advantage of providing representative information on ITH at higher resolution level [135]. CTC isolation appears technically challenging considering their extremely low proportion in the bloodstream (0.1–10 CTCs per mL of blood, representing about 1:10^6^–1:10^8^ of all blood cells) [135,136,146]. Current enrichment methods take advantage of CTCs physical (size, deformability, density and electric charges), biological features (expression of epithelial cell markers and lack of CD45 staining) or both, to specifically isolate CTCs from blood cells [147]. The CellSearch system was notably the first to receive FDA-approval technique to detect CTCs (EpCAM+, Cytokeratins+, CD45−) in patients with metastatic breast, prostate or colorectal cancer [148]. However, to date, no/only a few markers were identified as being exclusively present in tumours, allowing some cases of false results by CTC approaches [149].

#### 3.4.2. ctDNA

Cell-free DNA (cfDNA) is shed in body fluids by malignant and non-malignant cells through different biological processes, including apoptosis, necrosis and spontaneous active release (incorporated or not in extracellular vesicles or nucleoproteic complexes) [150]. CtDNA represents a small fraction of cfDNA that originates from tumour cells located in primary or distant tumour sites as well as CTCs [151]. CtDNA is characterised by small fragment length (around 146 bp) and contains tumour-specific genetic and epigenetic alterations. In the last few years, there has been an extraordinary enthusiasm in the use of cfDNA-based testing for cancer diagnosis and prognosis, treatment decision making, treatment response monitoring, clonal evolution tracking and early detection of resistance mechanisms [134].

In the Rep-Seq study, the clinical interest of plasma samples drawn at different time points was evaluated to delineate ITH in ccRCC compared to Rep-Seq and multi-region sampling [111]. Although it was not the main objective of the study, they reported a lower reproducibility between plasma samples (only 24% of similarity between pairwise samples) compared to other approaches. Given the temporal evolution of the cancers, heterogeneity was expected between the different time points; however, even samples from close time points presented disparate mutational landscapes. Inferring the clonal structure of tumours based only on plasma samples seems challenging, as ctDNA investigations missed a high number of events, including truncal variants. Yet, liquid biopsy based on ctDNA profiling holds great promise for the longitudinal monitoring of tumour markers and the detection of minimal residual disease (MRD).

In the same way, the NSCLC TRACERx study aimed at assessing the clinical utility of ctDNA to track cancer evolution in 100 patients with resectable Non-Small Cell Lung Cancer (NSCLC) [152]. Multi-regions of NSCLC tumours were first analysed by exome sequencing to reveal the phylogenetic tree of each tumour. Then, patient-specific multiplex PCR NGS assays were designed to follow, with ctDNA, a limited number of clonal and subclonal variants per patient. Almost half of the patients presented positive pre-surgery plasma samples with at least two variants detected. A total of 94% of the clonal variants and 68% of the subclonal variants targeted by the custom-made panels were retrieved in these samples. The VAF of the clonal variants were found higher than those of subclonal ones and correlated well with the tumour volume. The probability to detect subclonal variants in ctDNA samples increased with the spread of these variants in the different tumour regions. The characterisation of pre- and post-surgery plasma samples from 10 relapse-free patients and 14 patients with clinically confirmed relapse demonstrated that subclonal variants were detected more frequently in patients who develop tumour relapse (93%) than in relapse-free patients (10%). Variants were detected in plasma much earlier than is possible to confirm tumour relapse by current radiological imaging procedures (mean interval of 70 days between ctDNA detection and positive CT imaging). CtDNA profiling also helps to early identify resistance to adjuvant chemotherapy. Subclone variants in charge of metastasis spreading were successfully detected in plasma and can be tracked through iterative plasma samples. These results confirmed the observations made by Litchfield et al. [111] and foster the use of highly sensitive ctDNA-based custom-targeted panels for prognostic and resistance prediction purposes.

One challenge of using plasma samples in cancer care is the fragmentation of ctDNA molecules and their high dilution in cfDNA shed by non-malignant cells (representing a fraction as low as 0.01% of the total cfDNA [153]). Recent progress has been made in order to improve the detection performance of ctDNA-based approaches through the development of novel ultra-sensitive techniques or in silico error-suppression tools [154]. Some groups also proposed to more easily discriminate ctDNA from total cfDNA based on the profiling of ctDNA-specific features (methylation profile and fragment length) [155,156]. It also appears difficult in some cases to distinguish tumour-specific signals from physiological events that can appear with ageing. Almost 60% of healthy individuals harboured at least one non-synonymous alteration with similar VAF in both ctDNA and matched blood cell DNA [157]. Clonal haematopoiesis of indeterminate potential (CHIP) is an age-dependent biological process in which somatic mutations occur in hematopoietic progenitor cells and drive clonal expansion [158]. CHIP confers an increased risk of haematological malignancies and cardiovascular complications. CHIP can affect genes coding epigenetic modifiers (*DNMT3A*, *TET2*, *ASXL1*), spliceosome proteins (*SF3B1*, *SRSF2*), DNA damage response regulators (*TP53, PPM1D*) or cell signalling pathway actors (JAK2, GNAS) [159]. In such a way, CHIP can be easily confounded with mutations from tumour origin, especially as the exposure to some anticancer treatments (radiation, carboplatin, topoisomerase II inhibitors) favours the selection of clones with mutations in DNA damage repair genes in the context of CHIP [160,161].

## 4. Concluding Remarks and Perspectives

Tumour heterogeneity appears multifaceted and pervasive across cancer types and limits the diagnosis, prognosis and predictive value of tumour biomarkers. Although all levels of tumour variability lead to challenges in oncology, ITH is of particular interest considering its major consequences on cancer progression, metastatic dissemination, targeted therapy failure, drug resistance and recurrences. The clinical evaluation of tumour diversity should be a prerequisite for treatment-decision making; however, the information obtained routinely from a single-site tumour tissue biopsy is not sufficient to fully reflect the bulk tumour and its heterogeneity. Novel approaches are emerging to better capture ITH. They present differential advantages and limitations that have to be considered in order to select the most appropriate strategy depending on the clinical and biological context. Single-cell approaches can reliably define extensive tumour taxonomy but suffer from their cost and technical challenges. Multi-region sequencing represent appealing approaches to decipher the clonal architecture of the tumours and understand the biology of metastasis; however, Rep-Seq appears as a much more practical alternative for clinical routine. Liquid biopsy offers the advantages to more easily reveal spatial but also temporal ITH. The development of patient-specific cfDNA-based targeted assays notably holds great promise for the longitudinal tracking of genomic evolution during the course of the disease at more moderate cost.

## Figures and Tables

**Table 1:** Examples of tumour microenvironment (TME) heterogeneity and their consequences on cancer development.

TME Cell Types	TME Heterogeneity	Consequences on Tumour Development	References
Cancer-associated fibroblasts (CAFs)	CAFs differ by their origins (cellular precursors and cellular locations) and their marker expression profiles. CAFs subgroups are differentially expressed depending on the cancer types.	CAF subsets display opposite functions in cancers: some favour cancer development through the promotion of angiogenesis, metastasis and drug resistance, while others exhibit tumour-suppressor properties by contributing to growth inhibition, immune surveillance of the tumour and drug sensitivity.	[52]
Immune cells (macrophages, dendritic cells, mast cells, natural killer cells, B and T lymphocytes)	Variable levels of immune infiltration are observed in tumours depending on cancer types and subtypes. The immune cell composition (innate/adaptive immune cells, immune cell types) also differs between tumours.	Numerous studies report the interest of tumour-infiltrating lymphocytes (TILs) as a major prognostic marker in diverse cancers. High density of CD8+ T cells in tumours is strongly correlated with good prognosis, while high regulatory T-cell (Tregs) infiltration was associated with early recurrence and poor outcomes. In the same way, high density of NK cells in tumours was shown to predict good patient survival. Tumour-associated macrophages and neutrophils promote tumour cell plasticity and cancer stem cell phenotype, notably though the secretion of specific cytokines.	[53,54,55]
Tumour endothelial cells (TECs)	TECs show differences in terms of origins, morphology, structure, functions and marker expression. TECs derived from highly metastatic tumours harbour more cytogenic abnormalities and proangiogenic properties than those from tumours with low metastasis. TECs from tumours with high metastatic potential display a stem cell-like phenotype with the remarkable capacity to form spheres.	The overexpression of adhesion molecules in TECs allows cancer cell extravasation and metastasis spreading. TECs can secrete angiocrine factors at various levels that contribute to cancer cell proliferation, migration, invasion and angiogenesis. TECs also contribute to the emergence of drug resistance by increasing the expression of ATP-binding cassette transporters or helping tumour cells to switch to resistant phenotypes. TECs also modulate cancer immune surveillance by secreting growth factors that inhibit immune cells homing and induce the apoptosis of activated CD8+ T cells. TECs expressing PD-L1 marker hamper T cell activation.	[56,57,58]
Extracellular matrix (ECM): collagens, proteoglycans, fibronectin, elastins, laminins, hyaluronans	Proportion of ECM in tumours, ECM composition, architecture and posttranslational modifications are highly variable from a tumour to another.	Increased amounts of collagens in ECM of pancreatic cancers is associated with poor prognosis and chemoresistance. The expression levels of certain collagen isoforms (notably increased Col I levels and decreased Col IV levels) are correlated with stage of cancers and poor prognosis. Enhanced laminin expression and its anarchic distribution as well as high hyaluronic acid levels are correlated with poor clinical outcomes. Abundant and rigid ECM in tumours can act as a barrier and protect tumour cells from therapeutic agents. The stiffness of ECM and its enrichment in hyaluronic acid and Col I isoform drive epithelial-to-mesenchymal transition and promote metastasis and drug resistance.	[59]
Cancer-associated adipocytes (CAAs)	Less is known about the heterogeneity in the adipocyte part of the ECM. CAAs are characterised by irregular morphologies with decreased lipid content and reduced differentiation marker expression compared to normal mature adipocytes.	Growing evidence highlight the role of CAAs in the development of certain tumour types. CAAs interact with cancers cells and induce the reprogramming of their energy metabolism, the development of chemoresistance and the secretion of adipokines that modify the behaviour of tumour cells.	[60]
